# Molecular Handoffs in Nitrergic Neurotransmission

**DOI:** 10.3389/fmed.2014.00008

**Published:** 2014-04-10

**Authors:** Arun Chaudhury

**Affiliations:** ^1^Department of Surgery, Brigham and Women’s Hospital, Harvard Medical School and VA Boston Healthcare System, Boston, MA, USA

**Keywords:** nitrergic, neurotransmission, shank, autism, functional bowel disorders, LC8, PSD95, inhibitory neurotransmission

## Abstract

Postsynaptic density (PSD) proteins in excitatory synapses are relatively immobile components, while there is a structured organization of mobile scaffolding proteins lying beneath the PSDs. For example, shank proteins are located further away from the membrane in the cytosolic faces of the PSDs, facing the actin cytoskeleton. The rationale of this organization may be related to important roles of these proteins as “exchange hubs” for the signaling proteins for their migration from the subcortical cytosol to the membrane. Notably, PSD95 have also been demonstrated in prejunctional nerve terminals of nitrergic neuronal varicosities traversing the gastrointestinal smooth muscles. It has been recently reported that motor proteins like myosin Va play important role in transcytosis of nNOS. In this review, the hypothesis is forwarded that nNOS delivered to subcortical cytoskeleton requires interactions with scaffolding proteins prior to docking at the membrane. This may involve significant role of “shank,” named for SRC-homology (SH3) and multiple ankyrin repeat domains, in nitric oxide synthesis. Dynein light chain LC8–nNOS from acto-myosin Va is possibly exchanged with shank, which thereafter facilitates transposition of nNOS for binding with palmitoyl-PSD95 at the nerve terminal membrane. Shank knockout mice, which present with features of autism spectrum disorders, may help delineate the role of shank in enteric nitrergic neuromuscular transmission. Deletion of shank3 in humans is a monogenic cause of autism called Phelan–McDermid syndrome. One fourth of these patients present with cyclical vomiting, which may be explained by junctionopathy resulting from shank deficit in enteric nitrergic nerve terminals.

## Mere Localization of nNOS in Prejunctional Nerve Terminals is Inadequate for Nitric Oxide Synthesis

Defective neuromuscular transmission is a major pathophysiological basis for gastrointestinal motility disorders (1–3). Large repertoire of these disorders results from defective inhibitory neurotransmission that involves release of nitric oxide from prejunctional nerve terminals of motor neurons that traverse the smooth muscle layers of the gut (4–7). Failure of nitric oxide synthesis, the major inhibitory neurotransmitter, results in a phenotype that involves complete failure of gut smooth muscle relaxation (8). Though human biopsy samples of functional bowel disorders are difficult to obtain for obvious reasons, animal studies have provided ambiguous results regarding concentrations of nNOS in nerve terminals in pathological states (9, 10), which phenotypically manifest as either failure of relaxation of intestinal smooth muscles or varying degrees of impairment of gastrointestinal transit (11, 12). However, recent studies have provided evidence that mere presence of nitric oxide synthesizing-enzyme neuronal nitric oxide synthase (nNOS) in the nerve terminals may not be adequate for inhibitory nitrergic neuromuscular transmission in the gut (11, 12).

Nitric oxide-mediated neurotransmission, the main basis for oro-aboral movement of intestinal luminal contents, may be disrupted due to several factors, including (i) transcriptional blockade of genomic nNOS synthesis (13, 14), (ii) deficit in specific splice variants like nNOSα, because these splice variants have the capability to undergo lipidic modification to remain membrane-associated through N-terminal PDZ-interacting domain (15, 16), (iii) defect in allosteric proteins and cofactors like tetrahydrobiopterin (BH4) and LC8 (15, 17–20), (iv) defects in dimerization (16, 18, 21, 22), and (v) defective transport of nNOS within the nerve terminals due to cytoskeletal abnormalities, which do not favor enzymatic synthesis of nitric oxide (11, 12, 23).

Diverse organ systems reveal that nNOS remains membrane-bound during enzymatic synthesis, suggesting that membrane localization of nNOS may be critical for enzyme action in a physiological context (5, 24–33). Evidence has suggested the role of motor proteins like myosin Va in transposition of nNOS within the nerve terminals to the membranes to facilitate nitrergic neurotransmission (11, 12).

## Possible Role of Actin Cytoskeletal Barrier in Nitric Oxide Synthesis during an Action Potential

A thesis is proposed here, based on rational argument that depletion of the cytoskeletal organizer protein shank3 may result in defective nNOS membrane localization, resulting in defective nitric oxide synthesis. nNOS is a water soluble protein, but a portion of nNOS within nerve terminals remains membrane-bound due to its ability to undergo lipidic interaction with palmitoyl-PSD95 (15, 34). Membrane-bound nNOS may be at an optimal cellular localization for nitric oxide synthesis, possibly due to proximity to calcium ion channels (12). Myosin Va facilitates cytosolic transport of nNOS to the subcortical region of the nerve terminal that is rich in actin (12). Actin meshwork has been reported to provide a physical barrier to vesicles involved in neurotransmission (35–44). Specific dynamics regarding correlation of synaptic activity and reorganization of cortical actin has been examined in some neuronal systems (39, 45–48), but has not been tested in enteric nerve terminals.

The critical role of filamentous actin in determining the extent of dynamic reorganization in postsynaptic density (PSD) molecular composition is being increasingly recognized (49–52). It is not known whether actin network may provide a barrier to diffusion of non-vesicular neurotransmitter synthesizing enzymes like nNOS, but recent evidence suggests that the cytosolic streaming of water soluble molecules is not a chaotic stochastic event (53), but rather relies on the cytoskeletal machinery like myosin Va and actin for specific domain localization (11, 12, 54). Recently, the role of rare actin mutations in refractory constipation has also been recognized (55, 56).

## Possibility of “Shank” Proteins as Organizational Unit in Enteric Inhibitory Junctions: Molecular Exchanges during Nitrergic Synthesis

In excitatory synapses, the PDZ-domain-containing scaffold proteins PSD95, along with the shank family form a bilayer protein network below the postsynaptic membrane, which is bridged by guanylate kinase-associated protein (GKAP) (57). Shank protein has three different isoforms: shanks1, 2, and 3 (58, 59). Shank-family scaffolds are further linked to actin filaments via cortical-actin-binding protein (cortactin) (60). Thus, these shank proteins form sheets that make a synaptic platform (61, 62). Depletion and redistribution have been shown for ProSAP2/shank3 in PSDs of cultured neurons, an observation which was independent from protein synthesis or degradation and could be enhanced by electrophysiological stimulation (63). Whether such laminar organization occurs in enteric nitrergic nerve terminals is not known. Importantly, scaffolding proteins like PSD95, which are normal constituents of postsynaptic compartments, may also be present in presynaptic region, including enteric nerve terminals (15, 64).

Myosin Va has been shown to interact with nNOS via DLC8 (dynein light chain, 8 kDa MW) (12, 20, 65). LC8 acts as multiple cargo adapters and provides a hub for protein homo- and heterodimerization (66, 67). LC8, also called DLC8, has been reported to bind to presynaptic components like bassoon, which form cytomatrix of the active zone (66). LC8 has also been reported to associate and form macromolecular complex with shank (68).

Initial evidence has suggested the plausible existence of an “active zone” for nNOS in the membrane of these nerve terminals (15). nNOS is tethered to the nerve terminal membrane via the PDZ-rich protein PSD95 (15). PSD95, apart from its PDZ domains, also have other protein domains like SH3 and guanylate kinase (69, 70). nNOS may undergo molecular exchange in the region of the cortical cytoskeleton in which acto-myosin Va-bound nNOS initiates association with shank, a cortical actin-bound scaffolding protein.

**Figure 1:**
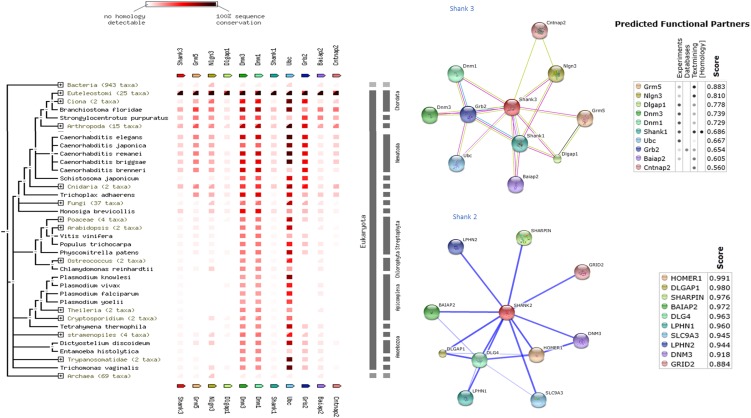
**Shank proteins are widely distributed in nature in both prokaryotes and eukaryotes**. Left panel shows protein association of shank3 across animal and plant kingdom. The interactive analyses were performed using STRING 9.1 software (71). Right panel shows protein-association partners of mouse shank3 (performed with STRING9.1) and human shank2 (performed with STITCH3.1) (72). Protein interaction partners reveal components of both postsynaptic (inotropic glutamate receptor GRID2, homer, GKAP, PSD, neuroligin) as well as presynaptic compartments (latrophilin, dynamin). Note that scaffolding proteins like PSD95 are not restricted to the postsynaptic compartment but are present presynaptically in enteric nerve varicosities. Note that shank3 and shank2, proteins that are both present in nitrergic myenteric neurons, have different binding partners. This may have an implication for distinct functions of shank3 and shank2 in nitrergic neurotransmission.

**Figure 2:**
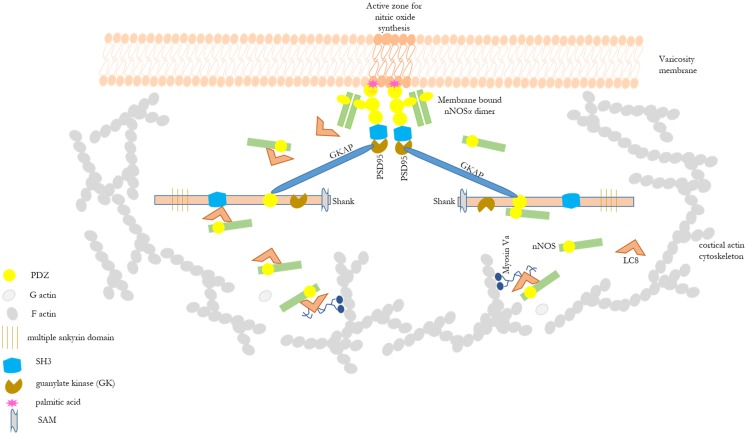
**Cartoon depicting possible role of shank in shuttling of cytosolic nNOS to the nitrergic nerve terminal membrane**. This depicts the basis of the hypothesis of possible role of shank in nitrergic neurotransmission. Different scaffolding proteins are shown with the modular domains without appropriate scale to the full length of the proteins. Details of these modular domains are described in Kim and Sheng (57). nNOS, via interaction with LC8 or PDZ-domain-based interaction, may be shuttled from acto-myosin Va in the cytosol through the actin cortical barrier by shank and GKAP. Shank has multiple protein interaction domains like ankyrin domains, SH3, PDZ, and SAM. Static protein interactions are planned to be examined to test whether such exchanges takes place in enteric nerve terminals. By these mechanisms, shank may play a significant role in nitric oxide synthesis and nitrergic neuromuscular neurotransmission. nNOS–LC8–myosin Va and nNOS–palmitoyl-PSD95 interactions have been demonstrated in enteric nerve terminals (12, 15).

Shank protein has different modular domains like multiple ankyrin repeats, SH3, PDZ, and sterile alpha motif, which all can function as protein interaction units. These aspects have been reviewed in details elsewhere (57). Figure 1 is a STRING analyses that shows shank proteins are widely distributed in nature in both the plant and animal kingdom. Furthermore, curated analyses show that shank3 and shank2 interact with multiple proteins that are known to be present and functional in both postsynaptic and presynaptic compartments. In the nitrergic nerve terminals, nNOS may bind to PSD95 in the membrane from shank via transposition through GKAP. PDZ-domain-mediated transfer of these proteins or molecular exchange via LC8 may occur at the cortical cytoskeleton of the enteric nerve terminal periphery. The logistics of this hypothesis is represented in a cartoon (Figure 2). These molecular exchanges may be viewed by time-lapse NMR experiments or by live imaging of enteric varicosities with evanescent microscopy or fluorescence correlation spectroscopy.

**Figure 3:**
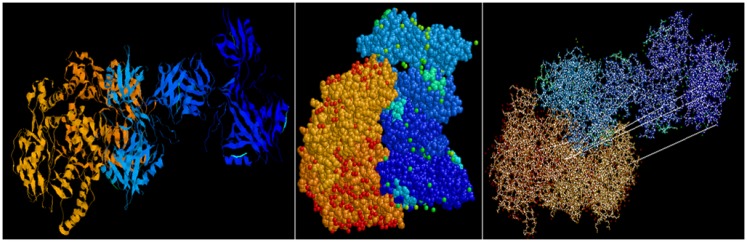
**Predictive bioinformatic analyses shows potential interaction between shank3 and nNOS**. Rat proteins (PDB: 3QJN, shank3; 1OM4, nNOS) were queried for interaction using Patchdock. Refinement of solutions was performed using Firedock. Protein interaction predictions were performed based on van der Waals and electrostatic interactions between the 3D structures. Prediction of structural alignment based on lowest energy levels were visualized for ribbons and ball and stick structures using RASMOL and depicted here from different angles. Patchdock software uses surface configurations of folded proteins to predict interactions using computer vision technology (74–76) and is currently being used in neuroinformatics (77).

**Figure 4:**
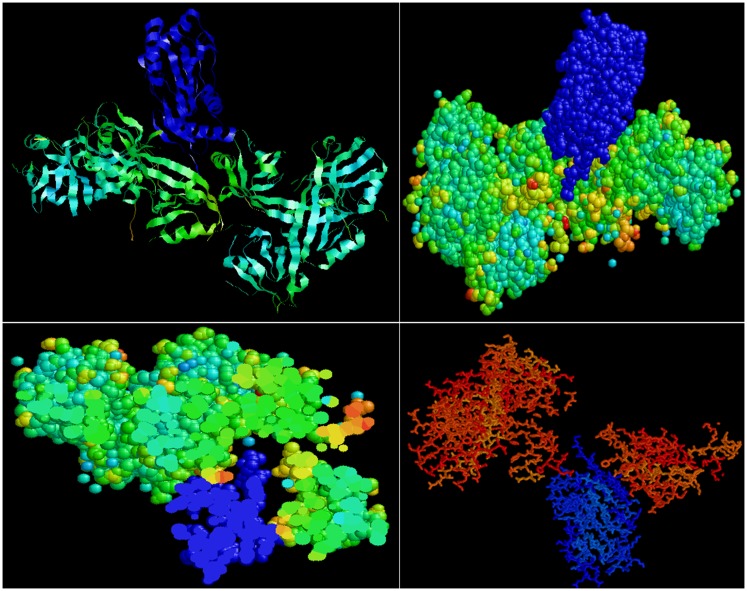
**Predictive bioinformatic analyses shows potential interaction between shank3 and LC8**. Rat proteins (PDB: 3QJN, shank3; 1F96, nNOS-bound LC8) were queried for interaction using Patchdock. Refinement of solutions was performed using Firedock. Protein interaction predictions were performed based on van der Waals and electrostatic interactions between the 3D structures. Prediction of structural alignment based on lowest energy levels was visualized for ribbon and ball and stick structures using RASMOL (78) and shown in upper panels. In the lower panels, note the groove in the shank in which the LC8 (blue) fits. Such conformation may offer dynamic stability to shank–LC8–nNOS complex during transport at the periphery of nerve terminals.

Predictive *in silico* analyses shows that shank3 has the potential to interact with both nNOS (Figure 3) and LC8 (Figure 4). This makes it likely that LC8 acts as an exchange factor that helps transcend sub-terminally located nNOS through the actin meshwork to membrane-bound PSD95. Dialyzates of membrane and cytosol have shown that membrane fractions of enteric nerve terminals lack LC8 (20). Proteomic analyses have shown that LC8 can bind to actin (73). It is thus likely that LC8, shank, and GKAP mediates transfer of nNOS in the subcortical zone of the nerve terminal, and that the subcortical zone of the nerve terminal is a critical zone for nitrergic neurotransmission. PSD95–nNOS complex may be formed in the cytosol but it is probably not feasible for this macromolecular complex to be transported to the membrane. Rigidly bound nNOS to PSD95 will not favor efficient neurotransmission. This mainly results from the state of the membrane potential in the region of the nerve terminal, which imperatively has to be closely correlated with the activity state of the nNOS enzyme. The loose associations between nNOS and LC8, myosin Va, PSD95, and possibly shank and GKAP add to the complexity of regulation of how nNOS is transported within the terminal but probably favor a state of switching between active and inactive enzyme function. This toggle nature is important for start and stop of nitrergic neurotransmission with classical Sherringtonian concepts.

**Figure 5:**
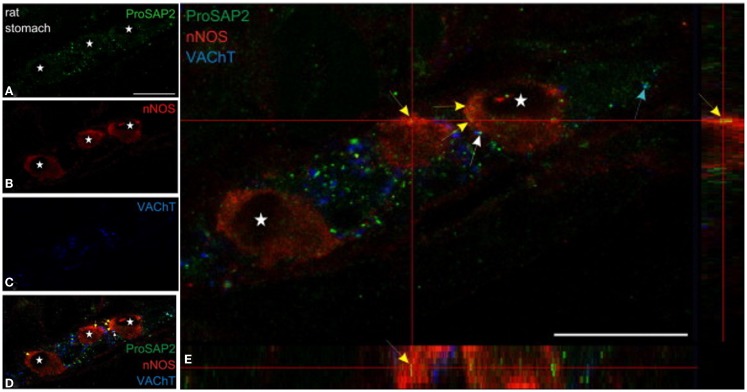
**Shank3 is present in soma of myenteric nitrergic neuron**. Note that shank3 has speckled appearance in cell periphery **(A)**. **(B–D)** shows staining for nNOS, vAChT and colocalization of the three proteins. In **(E)**, higher magnification view shows the speckled appearance of shank, which is still seen as diffuse distribution across the cell body, but it is not clear whether the distribution is only restricted to the surface. The higher power image shows diffuse distribution of nNOS in the both the cell cytosol and membranous region, colocalized with the submembranous location of shank3. Note that shank3 localization in nitrergic nerve terminals has not been examined. Modified with permission from Raab et al. (79).

Both shank3 and shank2 has been reported to be present in nNOS-positive myenteric neuronal cell body (79) (Figure 5). Shank protein presence has been described in the gut (80, 81), though it has not been examined specifically in the nitrergic nerve terminal. Messages for shank-interacting proteins like SHARPIN have been described from entire gut muscle extracts of wild-type mice (82). Testing important components of the role of shank in nitrergic neurotransmission are current goals of the laboratory.

## Shank Proteins may Play Significant Function in Enteric Nitrergic Neuro-Smooth Muscle Neurotransmission by Facilitating Transfer of Cytosolic nNOS to Membrane through the Cortical Cytoskeleton

Intriguingly, myosin Va-associated scaffolding proteins like “shank” have been reported to be depleted in monogenic conditions that result in manifestation as pervasive neurodevelopmental disorder (PND) (83). For example, 25% of patients with the rare condition Phelan–McDermid syndrome report refractory and cyclical vomiting (84–87). Chromosome mutations like deletions are seen in chromosome 22 in this syndrome (del22q13.3) (88, 89). This results in inhibition of synthesis of the protein shank3 (90). Shank proteins [Src-homology domain 3 (SH3) and multiple ankyrin repeat domains], including shank3, are known protein interaction partners of myosin Va (68, 91–94).

Gastrointestinal motility disorders affecting both the proximal and distal portions of the gut affect quality of daily life in both children and adult subjects with autism spectrum disorders (ASD) (95–97). These gastrointestinal motility problems manifest as dysphagia, achalasia, refractory or cyclical vomiting, acid reflux, gastroparesis and defect in gastric emptying, intestinal stasis and pseudo-obstruction, and chronic constipation (86, 98–106). Dysphagia in these patients, gastroesophageal reflux, chronic vomiting, or encopresis and chronic constipation are often misconstrued in the clinical setting as behavioral issues, rather than an organic problem (107–109).

In diverse systems, myosin Va–LC8–nNOS have been shown to form complexes with shank–GKAP–PSD95 (68, 73, 110). This important issue merits examination in enteric motor terminals. Shank has also been reported to associate with cell adhesion molecules like neuroligins in the postsynaptic compartment. Changes through neuroligin–neurexin signaling have been proposed in the presynaptic compartment during neuronal activity (111, 112). Additionally, neuroligin defects have been suggested as a pathophysiological basis for defective gastrointestinal neurotransmission in autism (113). Though NO signaling may not be spatially localized because of the very high diffusion coefficient (114, 115), specific role of cell adhesion molecules in nitrergic neurotransmission has not been examined in details.

Shank2 may also play a role in nitrergic neurotransmission in enteric neuro-smooth muscle junctions. Shank2–guanine nucleotide exchange factor ArhGEF interactions has been reported (116). Deletion of ArhGEF in mice has been shown to develop esophageal achalasia (117, 118). Both shank2 and shank1 mutations have also been reported recently to present with autism features (88, 119–121). The role of shank2/shank1 in enteric nitrergic neurotransmission may also be significant, though the presence of shank1 in nitrergic myenteric neurons was not reported in the study by Raab et al. (79).

## Shank Knockout Mice may Provide Insights into Mechanistic Basis of Cyclical Vomiting

Though the experiments of dynamically examining molecular exchanges by real-time live imaging may not be easily accomplished without access to high resolution microscopy, protein association studies by imaging proximity ligation assay (PLA) (12, 122) may be utilized to obtain a snapshot of static interactions in the cell periphery. Nitric oxide production assays by KCl stimulation of diaminofluorescein (DAF)-loaded enteric varicosities may be used to examine deficiency of nitric oxide production in shank3 knockout mice. Shank 3^exon4–9^ homozygotes result in nearly complete loss of shank3a and b isoforms (123). It has been reported that these shank3 knockout mice manifest behavioral patterns of autism (123–126). Defective nitrergic neurotransmission due to shank deficiency may impair gastric emptying. These studies shall provide critical insights into the molecular pathology of refractory gastrointestinal motility disorders like cyclical vomiting in patients with ASD.

Insights into molecular pathogenesis shall set the stage for long-term investigations into designing rational pharmacological targets for addressing these conditions. The gastrointestinal symptoms may severely affect nutrition in ASD patients with already compromised social communication skills, so state-of-the-art management for gastrointestinal problems is much needed. Gastrointestinal motility problems in these patients are a cause of suffering for the patients, as well as challenging issues for their caregivers including parents. Virtually nothing is known about the mechanisms underlying these disorders. This review argues based on incipient evidence from CNS neuropathology that because synaptopathy is a major underlying pathophysiology of ASD (112, 127–129), the motility problems of slowed gastrointestinal transit possibly result from defective junctional neuromuscular transmission, for example, through defects in nitric oxide-mediated neuro-smooth muscle transmission.

If defects in shank proteins are detected as a cause for impairment of nitrergic neurotransmission, then methods for pharmacological management for treating these disorders, such as replacement of shank proteins that are being reported for management of autism (130–132), may provide benefits for gastrointestinal symptoms as well. Shank proteins are known to respond to the enteric-specific neurotrophic factor GDNF via Ret tyrosine kinase signaling (133). These neurotrophic factors may also impact on management of gastrointestinal motility problems that may result from defective shank signaling. This review also supports the rationale of examining shank proteins in impairment of nitrergic neurotransmission in other functional bowel disorders like irritable bowel syndrome and idiopathic gastroparesis.

## Conflict of Interest Statement

The author declares that the research was conducted in the absence of any commercial or financial relationships that could be construed as a potential conflict of interest.
